# The Design, Content and Delivery of Relationship and Sexuality Education Programmes for People with Intellectual Disabilities: A Systematic Review of the International Evidence

**DOI:** 10.3390/ijerph17207568

**Published:** 2020-10-18

**Authors:** Michael Brown, Edward McCann, Maria Truesdale, Mark Linden, Lynne Marsh

**Affiliations:** 1 School of Nursing and Midwifery, Queen’s University Belfast, Northern Ireland BT9 7BL, UK; m.linden@qub.ac.uk (M.L.); l.marsh@qub.ac.uk (L.M.); 2 School of Nursing and Midwifery, Trinity College Dublin, Dublin 2, Ireland; mccanned@tcd.ie; 3 Institute of Health & Wellbeing, University of Glasgow, Glasgow, Scotland G12 0XH, UK; maria.truesdale@glasgow.ac.uk

**Keywords:** intimate relationships, sexuality, programme content, programme delivery, intellectual disabilities

## Abstract

There is growing empirical evidence regarding the relationship and sexuality experiences and needs of children, young people and adults with intellectual disabilities. A total of twelve papers met the inclusion criteria regarding relationship and sexuality education (RSE) programmes specific to the needs of this population. The preferred reporting items for systematic reviews and meta-analyses (PRISMA) guidelines were followed and quality appraisal undertaken. The four themes identified were principles informing RSE programme development, design and content of RSE programmes, delivery of RSE programmes and evaluation of RSE programmes. The discussion presents areas that need to be addressed to ensure that people with intellectual disabilities, their families, carers and professionals are fully involved in the design and delivery of RSE programmes. Further research is required to identify the impact of the programmes and the sustained outcomes achieved. Recommendations are made regarding the activities required to enable the development of evidence-based and person-centred approaches to relationship and sexuality programmes.

## 1. Introduction

Sexual expression takes many forms and contributes significantly to an individual’s health and wellbeing. The World Health Organization (WHO) highlighted in 2015 that the expression of sexuality is a fundamental human right and is multifaceted. The expression of sexuality includes safe and pleasurable sexual experiences free from discrimination, violence and coercion [1]. Internationally, there is an increased focus on improving health and developing ways of addressing health inequalities [2,3]. Health promotion and health education is advocated as a means to enable individuals and communities to make improvements in general health [4,5]. In order to be effective, such programmes designed to effect change in behaviour and attitudes need to be informed by recognized theoretical and behaviour change models [6]. Specifically, in relation to sexual health, the importance of sexual health improvement and prevention activities are recommended [7,8]. From a sexual health perspective, there has been increased attention on issues including sexual transmitted infections (STIs) [9], HIV and AIDS [10], youth pregnancy [11], contraception [12] and gender-based violence [13]. For children and young people, relationship and sexuality education (RSE) programmes have been developed to address these concerns for the purpose of providing knowledge and information to ultimately lead to a satisfying and fulfilling adult life [14,15]. RSE programmes for typically developing children and young people aim to provide knowledge and skills regarding the development and maintenance of healthy intimate relationships. A recent systematic review of the content and delivery of relationship skills education programmes identified that most were designed to be delivered in a school setting by a class teacher. The review also highlighted that the programmes broadly focused on the interpersonal dimensions of relationships [16]. To this end, RSE programmes enable people to make informed decisions about their future relationships, develop resilience and know where to access help and support when necessary [17]. To achieve this objective, many countries have established structured RSE programmes that are embedded within the school curriculum that is intended to be inclusive of all young people [18,19,20].

An intellectual disability (ID) describes limitations in intellectual functioning which occurs before, during or shortly after birth and is life-long. An ID affects intellectual functioning and is referred to as a spectrum condition involving mild, moderate, severe and profound cognitive impairment. Intellectual functioning such as learning, problem-solving and judgement can be impaired as well as limitations in adaptive functioning in areas such as self-care and communication [21]. From a rights and quality of life perspective, there have been significant developments in the care and support of children and adults with intellectual disabilities (ID) over the past decades from predominately institutional to community focused care and support [22]. The primary policy intention being to enable children and adults with ID to develop more autonomy and make informed decisions about key aspects of their lives [23]. As with the typically developing, people with ID want meaningful relationships and desire sexual intimacy and to be sexually active [24]. As young adults with ID transition from child to adult services tensions are evident regarding the expression of their sexuality due to concerns regarding the risks associated with exploitation versus the right to experience relationships and intimacy [25,26]. However, concerns exist around an individual’s lack of knowledge about relationships and appropriate sexual behaviours [27]. There may also be limited awareness of the potential risks of exploitation and abuse, unintended pregnancy and HIV and STIs [28,29]. Other factors that impact upon their ability to from relationships are linked to lack of opportunity, social isolation and communication deficits [30,31]. 

RSE programmes have been developed to address the distinct needs of people with ID. From the available evidence, these programmes appear to include issues such as capacity and consent, anatomy and biology, contraception and sexual behaviours [20]. It is unclear if such RSE programmes have been developed around theoretical behaviour change models with explicit intended outcomes. Ultimately, it is necessary to identify if participating in an RSE programme enhances long-term decision making that impacts positively on relationships. It also remains to be established who is involved in the design and delivery of RSE programmes for people with ID and the extent to which an evaluative process is integral to the approach. Therefore, the aim of this systematic review was to identify the design, content, delivery and evaluation approaches used in RSE programmes for people with intellectual disabilities across the lifespan.

## 2. Methods

The objectives of the review were:

- to identify who is involved in the design and development of RSE programmes for people with intellectual disabilities,
- to establish the content of the RSE programmes,
- to identify who delivers the RSE programmes and
- to identify if evaluations have been undertaken of the RSE programmes.

Prior to commencing the systematic review, the PROSPERO and Cochrane databases were checked to identify if a similar review had previously been untaken. No such reviews were identified. The preferred reporting items for systematic reviews and meta-analyses (PRISMA) process was followed [32].

### 2.1. Search Strategy

#### Ethics Statement

The study is a systematic review of published research evidence therefore ethical approval was not required. An expert subject Librarian assisted with the literature searching. The databases searched were PsycINFO, CINAHL, MEDLINE, and ERIC. The key terms included (i) intellectual disabilities (ii) sexuality (iii) relationships (iv) education and training. To establish if additional relevant studies existed, we hand searched reference lists and also checked Google Scholar to identify any additional papers.

### 2.2. Inclusion and Exclusion Criteria 

Quantitative, qualitative and mixed methods studies were all eligible for inclusion in the review. Studies were included that:

- focused on the design, content, delivery or evaluation of RSE programmes specific to people with ID,
- utilized an empirical research design,
- were published in the English language.
- were published from inception to August 2020.

Studies were excluded that:

- did not focus exclusively on RSE programmes for people with ID,
- did not address the design, content, delivery or evaluation of RSE programmes specific to people with ID,
- were not empirical research,
- were not published in English and
- were grey literature and theses.

Following the search of the databases, studies were screened against the inclusion and exclusion criteria. Two reviewers screened the title and abstracts using the study inclusion criteria following the removal of duplicate papers. The reviewers retrieved and independently screened the full text papers. The reviewers then independently appraised the full text and completed an inclusion and exclusion checklist. Final agreement of the included papers was reached by consensus.

### 2.3. Data Extraction and Synthesis

Data were extracted on country, study aim, design, sample characteristics, study method, key findings and recommendations. The synthesis of the mixed methods research literature was conducted as part of the systematic review process [33]. The data were thematically analysed to identify the emergent themes across the included studies. Covidence was used to facilitate the review process [34]. Detailed and comprehensive coding and analysis was undertaken with the data verified by the research team. The identified concepts were grouped into themes to enable comparisons and differences to be established across and between the studies and the themes. To address potential bias, the final themes were discussed, verified and agreed by the research team [35].

### 2.4. Quality Assessment

The appraisal process involved two reviewers. The mixed-methods appraisal tool (MMAT) was used in the assessment of the quality of the included studies [36]. To determine quality, the appraisal questions were systematically applied and a category of ‘high’, ‘medium’ or ‘low’ assigned based on the evidence regarding specific criteria. Of the nine qualitative studies, set out in Table 1, eight scored ‘high’ [37,38,39,40,41,42,43,44] and one scored ‘low’ [45].

The three quantitative studies set out in Table 2 achieved ‘medium’ scores [46,47,48].

## 3. Results

**Figure 1:**
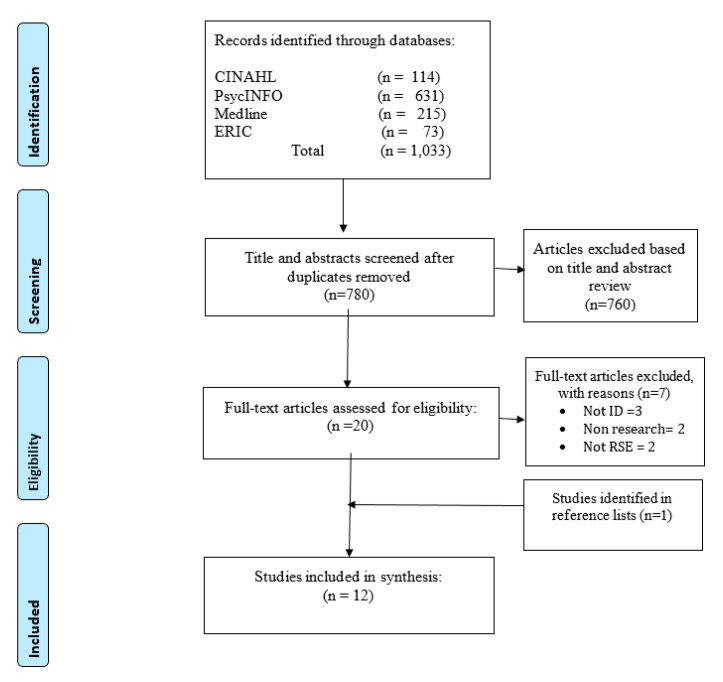
Preferred reporting items for systematic reviews and meta-analyses (PRISMA) flow diagram with search results.

The study selection process is set out in Figure 1, with the number of papers identified and included at each stage. Most of the full text articles were excluded as they did not report on the design, content, delivery or evaluation of RSE programmes specific to people with ID.

### 3.1. Study Characteristics

The 12 papers that met the aim of the review are set out in Table 3. Of the 12 included studies, 9 used qualitative designs and 3 utilised quantitative methods. A range of data collection approaches were used, including questionnaires, individual interviews and focus groups. The largest number of studies were conducted in South Africa (*n* = 3) and Sweden (*n* = 3). The others were undertaken in Australia (*n* = 2), Canada (*n* = 1), the Netherlands (*n* = 1), the United Kingdom (*n* = 1) and the United States (*n* = 1). The sample sizes ranged from 11 to 600 participants.

### 3.2. Data Analysis and Synthesis

Following the systematic analysis of the studies, four themes were identified: (i) principles informing RSE programme development; (ii) design and content of RSE programmes; (iii) delivery of RSE programmes; and (iv) evaluation of RSE programmes.

#### 3.2.1. Principles Informing RSE Programme Development

Arising from the current literature review is the need to ensure that RSE programmes for people with ID are informed by underpinning principles and relevant theoretical and behaviour-change models. The principles include parity of access to RSE programmes in keeping with those provided for typically developing children and young people [40,42]. Access to such RSE programmes needs to be viewed as a fundamental right to ensure that people with ID have access to evidence-based structured relationship and sexuality education that is appropriate to their specific needs [37,38,39,40,41,42]. The current research evidence highlights that existing RSE programmes lack recognised evidence-based models and frameworks. Therefore, the starting point to the development of RSE programmes relevant to people with ID is to ensure that they are built around and informed by relevant theoretical and behaviour change models [39,43]. In a Dutch study investigating five RSE programmes, the use of relevant theories and behaviour change models was found to be absent [43]. Research evidence also indicated that RSE programmes should be established for people with ID across the lifespan, commencing at an early age and continuing on into adulthood, and not delivered as a ‘one-off’ exercise [42]. The broad focus and intended purpose of some of the RSE programmes were to provide people with ID with the same opportunities as typically developing people. The broad focus being the provision of knowledge and skills to make informed choices and decisions to enable the development of positive sexual identities and benefit from healthy sexual relationships while reducing the possibility of harm [38,42,48].

#### 3.2.2. Design and Content of RSE Programmes

To be effective, RSE programmes require careful design to ensure that content is reflective of the needs and concerns of people with ID. Programmes should be designed around a structured evidence-based education framework and education delivery model and be adapted, where necessary at the point of delivery, to meet the needs of individual students [43,48]. Content should be evidence-based and designed by experts in the field with knowledge and experience of the relationship and sexuality needs and concerns of people with ID across the lifespan [37,45,46]. This is highlighted as important as the providers of some RSE programmes often relied on their own attitudes and values to guide content development [41,44,47]. In an attempt to overcome these issues, several studies identified the need for RSE programmes to be co-designed and co-produced in collaboration with people with ID to ensure they are reflective of their needs and aspirations [37,39,43,46,47]. As part of the co-design and co-production of RSE programmes, families of people with ID also need to be involved to ensure that their issues and perspectives are recognised and included [39,45,46,47]. This parental involvement is highlighted as important, given their concerns and the often-sensitive nature of pertinent issues, to ensure that the implementation of programmes is both effective and supported [40,46]. 

Some studies in this review detailed or recommended content for programmes based on the specific views of people with ID and of families and professionals. The suggested programme content varied depending on the concerns and priorities identified by the different groups. A total of five studies specifically referred to programme content from the perspective of people with ID [37,39,42,45,48]. In a study undertaken in the United States (US), young people with ID suggested that programmes should include a focus on intimate relationships and the acquisition of knowledge regarding sexuality and sexual expression [45]. In a Swedish study, young people with ID suggested content that included promoting a more affirmative and empowering approach towards the expression of sexuality and intimacy. Participants wanted more knowledge about sexually transmitted infections (STIs) and pregnancy concerns [39]. An Australian study described four educational sessions focusing on sex and relationships, rights and being safe and respectful relationships [37]. In one study undertaken in Kenyan schools, study participants highlighted that programme content needs to move beyond risk and harm and also include a focus on supporting human rights and addressing issues related to stigma and negative stereotyping of people with ID [42]. From a practical perspective, the participants recommended that programmes include health, personal hygiene, reproductive health and options regarding the expressions of intimacy [42]. A Canadian study involving young people with ID suggested that programmes include a focus on sexuality, sexual relationships and intimacy, as a means to promote choice and enhance positive sexual identities, another important facet related to recognising and discriminating between appropriate and inappropriate sexual behaviours and where to seek help [48].

From the perspective of parents of young people with ID, they expressed concerns about addressing the risk of sexual abuse and potential harm within programmes [45]. Contrastingly, the issues identified by teachers, health personnel and ID professionals include sex and interpersonal relationships, nutrition and physical activity, anatomy and physiology and reproductive health [45,46]. However, differing perspectives existed for some professionals regarding the inclusion of information on sexual harm prevention, health concerns and legal issues [45,48]. Other issues identified as necessary by health professionals included STIs, contraception and pregnancy issues and sex and interpersonal relationships [45,46]. For some teachers, RSE programme content should include bodily self-care; sexuality expression in sexual relationships; STIs, HIV and AIDS; contraception and pregnancy and online social behaviour [41,42,47].

#### 3.2.3. Delivery of RSE Programmes

From this review of the literature, no professional group emerged as the ‘natural’ one to deliver RSE programmes. People with ID have been identified as peer educators in the co-delivery of RSE programmes, however, further research is required on the effectiveness of this approach and the outcomes achieved [37,39,48]. To be effective, it is suggested that RSE programmes need to be fully integrated within the school curriculum for young people with ID [40,46]. Furthermore, one study suggested that RSE programmes should be delivered collaboratively between teachers, nurses and parents [46]. A proactive and positive approach was identified as important regarding programme delivery, involving key stakeholders, including young people with ID, parents and teachers [42,45,46,48]. Additional support was also identified as necessary to ensure that all young people with ID can fully engage in RSE progammes, supported by delivery guidelines to ensure consistency and access to tailored resources [38,39,40,42]. Two groups were identified who currently deliver programmes: nurses and school teachers. However, there are mixed views regarding the knowledge and skills required to confidently and effectively deliver RSE programmes [39,41,46]. Some teachers in one study were of the view that they had the relevant knowledge and skills but questioned if they were best placed to deliver RSE programmes [47]. In another study, teachers were of the view that there were gaps in their knowledge, notably around sexual health and cultural and religious aspects [41]. In another study, social care support workers questioned their role regarding sex education highlighting the possibility of role confusion [44]. Further education and practice development opportunities were identified as necessary to enable professionals to develop the necessary knowledge, skills and confidence in RSE programme delivery [42,46].

#### 3.2.4. Evaluation of RSE Programmes

To evidence their effectiveness, RSE programmes need to incorporate clear outcome measures and include formal evaluation, an integral element of delivery to identify impact and whether the intended outcomes have been achieved [41,42,43]. It is highlighted that programme evaluation and research should involve all key stakeholders including young people with ID and their parents [47]. Further, long-term follow-up is required to identify sustained behaviour change overtime [43]. Effective RSE programme evaluation is required to identify the impact by decreasing sexual abuse and exploitation [48]. While recommended in two studies, RSE programmes involving peer educators with ID need to be systematically and rigorously evaluated to identify their effectiveness and outcomes achieved [37,48]. One study suggested that the evaluation of RSE programmes also needs to identify the changing attitudes of communities towards people with ID and their right to equality to have relationships and express their sexuality [42].

## 4. Discussion

The twelve studies included in this systematic review were predominantly undertaken within the past ten years. One study was undertaken in Sweden and Japan in 1990 [46]. The research attention over the past decade may be due to several factors. The first may relate to the attention being given to the need for RSE programmes for all children and young people to ensure that they are equipped with the knowledge and skills to enjoy satisfying and fulfilling adult lives [49]. Another driver for the research focusing on people with ID may be due to concerns regarding the risks, such as exploitation and abuse, and wider sexual health concerns [28]. Three of the papers included in the current systematic review were conducted in Africa in response to concerns regarding sexuality and, more specifically, HIV and AIDS [40,42,47]. Evidence suggests that there are over five million people living with HIV in South Africa with the needs of people with disabilities, including those with ID, largely ignored. As a consequence, this may have influenced the need to conduct the three studies within Africa. Sweden, in contrast, has had a long history of providing mandatory sexuality education since 1955, which has evolved and is fully integrated in the school curriculum. Three of the studies were conducted in Sweden and are set within the wider context of the right to access sexuality education for all [39,41,46]. Therefore, the focus on RSE programmes from the perspective of people with ID may be driven by this rights and social inclusion agenda.

An important finding from the current systematic review relates to the evolving RSE programme research that identifies the design, delivery and outcomes achieved and their impact on behaviours over time. It is also evident that there is an absence of evidence-based RSE programmes that address the specific needs and concerns of people with ID across the lifespan, notably after the transition into adulthood. What is also required to be established is the evidence and processes utilised in the development of RSE programmes and who has the relevant knowledge, skills and confidence to effectively deliver such programmes. It is also yet to be established what the long-term benefits and outcomes are as a result of the existing programmes. This therefore indicates the pressing need for a policy focus, practice development and education initiatives to advance this agenda.

### 4.1. Policy

With the growing need to ensure that RSE programmes are available and accessible to all children, including those with disabilities or special needs, the UK Government have embedded RSE programmes into the school curriculum for all pupils in primary and secondary schools from autumn 2020 [17,50]. While the RSE needs of young people with ID are distinct and responses will inevitably change over time, the guiding policy is that they have the same fundamental right to access education programmes as typically developing children and young people, one of which is access to evidence-based RSE programmes [40,51]. It is evident from the findings arising from the current systematic review, that access to age-appropriate and evidence-based RSE programmes within the education system is required for young people with ID and that need continues into adulthood. An important finding from the current systematic review is that the focus of existing RSE programmes is on children and young people with ID. Only one study focused specifically on the RSE needs of adults with ID [37]. Therefore, future policy initiatives need to ensure that the needs of all people with ID are included, not only children and young people. Such programmes are required to prepare people with ID across the lifespan to form and maintain healthy relationships such as friendships, and sexual relationships including intimacy [17].

To date, and as evidenced in the current review, RSE programmes vary in content, with no ‘natural’ professional or parent emerging as best placed to deliver them. Rather, it is apparent that RSE programme design and delivery needs to be a collaborative approach in which young people and adults with ID, their parents and professionals all play crucial roles. The content of RSE programmes can be a contentious issue for some parents and professionals. However, it is important to note that concerns including consent, same-sex relationships and online presence such as sexting, pornography and cyberbullying feature in the recommended UK RSE programme content [17,50]. There is broadening of the traditional views and perceptions of what may constitute RSE programme content; it must be specific to the needs and concerns of children, young people and adults with ID. While it is possible to embed RSE programmes as a compulsory subject in the school curriculum, this is not the case for adults with ID and is an area that needs to be addressed. For children and young people with ID, parents remain the primary educator and therefore they need to be fully involved in the process [17]. It is also necessary to identify additional resources to support and enable conversations at home regarding relationships and sexuality issues thereby promoting the confidence of parents [50]. To be effective, young people with ID, parents and professionals must work collaboratively to maximise RSE programme content, delivery and evaluation.

### 4.2. Practice

From a practice perspective, this review highlights the need for professionals to develop the necessary skills and knowledge to deliver RSE programmes with confidence to young people with ID [42]. The prerequisite knowledge and skills are required to ensure that programme delivery responds to the specific needs of young people and adults with ID [43,44]. This is necessary to be responsive to different learning styles and needs. Young people and adults with ID need to be involved in all stages of the development of RSE programmes as the ultimate recipients [44,45]. The UK Government statutory guidance also highlights the need for professionals to work collaboratively with parents to facilitate communication, thereby helping to ensure that RSE programmes respond to and continue to meet the needs of the young person with ID and recognise their concerns [17].

The current review highlights the importance of formally evaluating the outcomes achieved following RSE programme delivery [48]. Evaluations need to be undertaken through the lens of people with ID to ensure RSE programmes reflect, respond and adapt to their specific concerns and priorities. These personal perspectives, either individually or as part of a wider group, can be used to inform the review and modification of RSE programmes, thereby ensuring they are contemporary [48]. Additionally, RSE programme reviews need to include the views of parents and professionals, thereby helping to ensure they remain contemporary and responsive to their concerns for their family member with ID, such as maintaining and sustaining positive and healthy relationships and minimising the potential for sexual abuse or unintended pregnancies [41]. To be effective, RSE training programmes must also enable professionals involved in their delivery to address cultural and religious beliefs that exist within different communities in a way that is relevant and acceptable [42].

### 4.3. Education

RSE programmes for young people with ID should be fully integrated within the school curriculum, be built upon education frameworks and effective teaching models and use evidence-based interventions to aid professionals in their delivery [52]. School teachers are one of the main sources of information for students, therefore, educating them in RSE programme delivery is necessary to promote the principles of inclusive education [44]. Education and training as part of general teacher preparation and continuing professional development (CPD) programmes should be developed and provided to improve the knowledge, skills and confidence of teachers to facilitate the effective delivery of RSE programmes [53]. It is important that teachers receive the appropriate training to be able to address issues related to culture and different religious beliefs [54]. This is necessary to support the adaptation of the content of RSE programme learning resources and materials to enable meaningful engagement and participation [20].

Health professionals require psychoeducation to develop their understanding of the importance of RSE for children, young people and adults with ID and recognise how the provision of RSE can help in reducing and preventing health and psychological problems across the lifespan [55]. In addition, as part of their general training or CPD all health care professionals require education about intellectual disabilities and their specific health needs [56]. Moreover, parents and families also have education and support needs that may enhance their understanding, attitudes and response regarding the changing needs of children with ID as they mature and develop into adulthood [57]. Some people with ID may experience challenges in forming and expressing their sexual identity [29]. Significant barriers to this include societal perceptions of people with ID and the impact of multiple disadvantages with regard to gender, sexuality and disability [58]. The removal of barriers through the provision of training and education can play a vital role in supporting young people and adults with ID to express their sexual identity and develop fulfilling relationships [24]. Efforts to educate the wider public about the relationships and sexuality rights of people with ID is also necessary to promote equality and inclusion [59,60].

## 5. Strengths and Limitations

A strength of the current systematic review is the identification of important issues regarding the design and development of RSE programmes relevant to the needs of children, young people and adults with ID. Attempts have been made to address the relationship and sexuality concerns of young people with ID, however, there has been a limited focus on extending these education initiatives and developments as they move into adulthood. Any RSE programme needs to be underpinned by relevant theoretical models to enable and sustain behaviour change. There is a need to evaluate outcomes and their long-term impact in influencing the relationship and sexuality experiences of people with ID across the lifespan. There is a lack of transcultural perspectives that needs to be recognised and responded to in RSE programme development. There were no multi-centre international research studies identified. The researchers sought to be rigorous in approach while conducting the review and acknowledge the potential for bias.

## 6. Future Research

Existing RSE programmes for both young people and adults with ID lack a theoretical basis [43]. Therefore, future RSE programmes need to be developed using systematic theories, models and evidence-based approaches and be fully evaluated to identify their impact and outcomes [42]. The use of multi-centred studies will determine the effectiveness of theoretically driven, structured RSE programmes that are specific to the distinct needs of young people and adults with ID and their families and professionals with a national, international and transcultural focus. Future RSE programme development needs to include children, young people and adults with ID in all aspects of their development. The concept of co-production should be at the core of all RSE programme design and development to ensure the needs and concerns of people with ID are integrated and accurately reflected [61,62]. A future research focus needs to identify the outcomes of RSE programmes that enable young people and adults with ID to develop positive sexual identities and decrease risk of sexual harm, unintended pregnancy and sexually transmitted infections [29]. There is also scope to undertake research studies that focus on the effects of religious and cultural beliefs and attitudes and the impact of RSE programmes for people with ID [63]. Prioritising research in these areas will help to address inequalities and enable young people and adults to make informed decisions about forming relationships and the expression of their sexuality.

## 7. Conclusions

It has become increasingly apparent through this systematic review of the research evidence that people with ID have distinct needs regarding their relationship and sexuality concerns. There is an evolving research evidence base regarding the design and delivery of RSE programmes to address their specific issues. While these developments are positive, it is important to recognise the limitations that exist regarding the RSE programmes currently available. It is apparent that any future RSE programmes must be designed around a recognised and evidence-based theoretical model that is effective in enabling and sustaining behaviour change. RSE programme content may be an area of contention and potential conflict, due to the potentially differing views of people with learning disabilities and their families. It is recommended that to be acceptable, people with learning disabilities, their families and professionals must all work collaboratively on the programme content design, which should also be informed by the research evidence base. It is necessary that all stakeholders are fully involved to ensure that the range of issues and concerns are fully reflected within the RSE programme. Failure to consider these important issues may result in poor uptake. The two key professional groups that emerged as those most commonly involved in the delivery of RSE programmes are school teachers and registered nurses. Some were of the view that they did not possess the necessary knowledge and skills to confidently deliver RSE programmes to people with ID. Therefore, to address these needs it is necessary to provide access to education and practice development initiatives that build their knowledge, skills and confidence. While it is apparent that there are RSE programmes being provided to some people with ID, it is yet to be established what their impact is in effecting long-term behaviour change. Arising from this, there is a pressing need to integrate clear outcome measures within all RSE programmes to enable the outcomes achieved to be identified. Additionally, there are no longitudinal studies in existence that identify the outcomes achieved regarding the development of positive and healthy relationships, intimacy and the expression of sexuality by people with ID. These are aims of RSE programmes and are therefore areas requiring further research attention.

## Figures and Tables

**Table 1:** Methodological quality of qualitative studies using MMAT (Hong et al., 2018).

Studies	Q1	Q2	Q3	Q4	Q5	Quality Score
Frawley & Bigby (2014)	Y	Y	Y	Y	Y	H
Lafferty et al. (2012)	Y	Y	Y	Y	Y	H
Löfgren-Mårtenson (2012)	Y	Y	Y	Y	Y	H
Louw (2017)	Y	Y	Y	Y	Y	H
Nelson et al. (2020)	Y	Y	Y	Y	Y	H
Phasha & Runo (2017)	Y	Y	Y	Y	Y	H
Schaafsma et al. (2013)	Y	Y	Y	Y	Y	H
Swango-Wilson (2009)	Y	CT	CT	N	N	L
Wilson & Frawley (2016)	Y	Y	Y	Y	Y	H

Y = yes, indicates a clear statement appears in the paper which directly answers the question; N = no, indicates the question has been directly answered in the negative in the paper; CT = can’t tell, indicates there is no clear statement in the paper that answers the question Critical appraisal questions were as follows: 1. Is the qualitative approach appropriate to answer the research question? 2. Are the qualitative data collection methods adequate to address the research question? 3. Are the findings adequately derived from the data? 4. Is the interpretation of results sufficiently substantiated by data? 5. Is there coherence between qualitative data sources, collection, analysis and interpretation?

**Table 2:** Methodological quality of quantitative studies using (MMAT) (Hong et al., 2018).

Studies	Q1	Q2	Q3	Q4	Q5	Quality Score
Katoda et al. (1990)	Y	CT	Y	N	CT	M
Louw (2014)	Y	N	CT	CT	Y	M
Murray (2019)	Y	CT	Y	CT	Y	M

Y = yes, indicates a clear statement appears in the paper which directly answers the question; N = no, indicates the question has been directly answered in the negative in the paper; CT = can’t tell, indicates there is no clear statement in the paper that answers the question. Critical appraisal questions were as follows: 1. Is the sampling strategy relevant to address the research question? 2. Is the sample representative of the target population? 3. Are the measurements appropriate? 4. Is the risk of nonresponse bias low? 5. Is the statistical analysis appropriate to answer the research question?

**Table 3:** Papers included in the review (*n* = 12).

Citation and Country	Aim	Design, Content and Delivery	Sample	Methods	Key Findings	Recommendations
Frawley & Bigby (2014) Australia	Identify the experiences of people with intellectual disability (ID) as peer educators in sexuality and relationship education.	Co-produced with people with ID Peer educators	Peer educators (*n* = 16)	Qualitative: interviews using thematic analysis	People with ID as peer educators acquire new knowledge and skills about relationships and available community resources and supports evident. Sharing their personal insights and experiences as a peer educator resulted in their greater empowerment and confidence.	Participating as a peer educator appears beneficial to individuals. Future work needs to focus on identifying the effectiveness of peer education and the outcomes for programme participants.
Katoda et al. (1990) Sweden	Identify the views of school nurses on health education and sexual relationships for young people with ID.	Health education and interpersonal relationships Delivered within regular curriculum	School nurses (*n* = 600)	Quantitative: questionnaire using descriptive statistical analysis	Swedish school nurses (47%) were more involved in delivering the programme compared to 1% of Japanese nurses. Nurses identified by parents as most appropriate to deliver sex education followed by teachers. Only 2% of nurse participants thought that nurses should have a lead role due to their limited knowledge of ID issues. 70% of Swedish participants thought nurses required education on ID compared to 91% of Japanese who stated they did not. Swedish nurses provided education on sex and interpersonal relationships, food and exercise and ‘our body.’	Sex and relationship education should be fully integrated with the school curriculum and delivered collaboratively by parents, teachers and school nurses. Specific teaching materials need to be developed with guidelines for parents, teachers and school nurses regarding their use.
Lafferty et al. (2012) UK	Identify the barriers to the delivery of relationship and sexuality education (RSE) for people with ID.	Interactive CD-ROM Sex, sexuality and relationships	Family carers, professionals and front-line staff (*n* = 100)	Qualitative: interviews and focus groups using thematic analysis	Main programme content related to the protection of vulnerable young people with ID and the lack of appropriate training, poor education resources and ‘cultural prohibitions.’ The barriers need to be identified, discussed and adequately addressed to improve RSE programmes’ content and delivery.	Training and information about RSE programmes are required. Risk management procedures need to be in place. RSE programmes should be available to support the empowerment of young people with ID.
Lofgren-Martenson (2012) Sweden	Explore the experiences of sex education programmes in young people with ID.	RSE education framework didactic delivery in gender segregated groups Content more focused on sexual risks	Young people with ID (*n* = 16)	Qualitative: interviews using thematic analysis	Current programmes focus more on sexual risk opposed to sexual pleasure and intimacy. There is a need for RSE education frameworks and teaching models to assist professionals to deliver RSE education programmes relevant to the needs of young people with ID.	Future studies should include young people with ID. There needs to be collaborators in the research to address different gender perspectives and co-create sex education programmes. Future studies should focus on learning strategies to reduce sexual risk behaviours and promote affirmative attitudes towards the expression of sexuality.
Louw at al. (2014) South Africa	Identify the views of teachers and childcare providers regarding sexuality, HIV and AIDS education in special needs schools.	Health education HIV/AIDS and sexuality education Collaborative approach by teachers and health professionals	Special school educators (*n* = 78)	Quantitative: questionnaire using descriptive and inferential statistical analysis	Teachers had high level of knowledge regarding the topic area and teaching sexuality education. However, some teachers questioned if they should be responsible for delivering RSE programmes. The personal attitudes and beliefs of teachers has the potential to influence teaching practice.	Policy research required on the impact and outcomes of RSE programmes. A proactive collaborative approach is needed to support RSE programme design and delivery. Tailored RSE materials are required for people with ID. Participatory action research is needed involving all key stakeholders, including young people with ID and their parents to identify the effectiveness of programmes.
Louw (2017) South Africa	Identify the experiences of teachers and school staff when delivering RSE programmes in special needs schools.	Sex education manuals Visual materials re sex and relationships School staff and parental involvement	Teachers (*n* = 68) School staff (*n* = 10)	Qualitative: questionnaire using thematic analysis	Students with ID have a fundamental right to receive RSE education relevant to their needs. Appropriate RSE curriculum is required and adequate support available to enable young people with ID to meaningfully engage and participate in RSE programmes.	Need up-to-date, evidence-based RSE programmes that are developed by experts in the field. This may address the possibility of teachers imposing their own values and beliefs. Parents need to be supportive of their children’s involvement in structured RSE programmes.
Murray (2019) Canada	Develop, deliver and evaluate a sex education programme for young people with ID.	Community development approach Sexuality, sexual relationships and intimacy Interactive approaches to learning	Young people with mild ID (*n* = 93)	Quantitative: questionnaire using descriptive statistical analysis	Reinforce the need for sexual health education for young people with ID and increase opportunities to develop healthy sexual relationships and intimacy. RSE programmes need to promote positive sexual identities and decrease risk of sexual harm.	Future research could utilise focus groups to more fully understand the perceptions of young people with ID regarding the education sessions. The benefits of a peer-to-peer model of education delivery needs to be researched.
Nelson et al. (2020) Sweden	Explore the experiences of teaching sexual and reproductive health to students with ID.	Sexual health and reproduction education Various teachers	Teachers (*n* = 10)	Qualitative: interviews and phenomenological analysis	Teachers are the main source of information for students. Teachers need to adapt content to student needs. Teachers lack knowledge and confidence regarding religion and cultural aspects and lack skills in sexual health issues.	Teachers need access to specific materials and resources. Teacher training programmes must address issues related to culture and different religious ideologies. An evaluation of learning outcomes needs to be undertaken.
Phasha & Runo (2017) South Africa	Identify the sexuality education needs of learners with intellectual disabilities in schools in Kenya.	Erratic and ineffective sexuality education. Mainly risky behaviours School, friends and mothers	Students with mild ID (*n* = 56)	Qualitative: interviews and focus groups using thematic analysis	Sex education is patchy with no formalised RSE programmes, resulting in a lack in ability to make informed decision regarding sex issues. RSE programmes need to educate regarding avoiding risky or dangerous situations. Content should be well structured to empower young people with ID and include anatomy, health, personal hygiene, reproduction and expressions of love. RSE programmes should begin at an early age.	Teachers require additional training regarding sexuality issues. Future research needs to address topics such as community attitudes towards young people with ID and their sexual and human rights, the benefits of training programmes for teachers, and evaluation of programmes undertaken.
Schaafsma et al. (2013) Netherlands	Explore the development of sex education programmes for people with ID.	Various teaching and learning methods adopted. Knowledge, teaching skills, tailoring, empowerment and enjoying sexuality.	Programme developers (*n* = 11)	Qualitative: interviews using content analysis	RSE programmes currently lack theoretical models and specific outcomes, and there is a need for systematic evaluations to identify behaviour change. RSE programmes need to include young people with ID in the development.	Future sex education programmes need to be developed using systematic theories, models and evidence-based approaches and be fully evaluated to identify their impact and outcomes.
Swango-Wilson (2009) USA	Identify the expectations and the development of a sex education programme.	Current materials too broad and overwhelming. Relationship development and skills for responsible sexual activity.	People with ID (*n* = 3), parents (*n* = 3), professionals (*n* = 6)	Qualitative: interviews using thematic analysis	Regarding RSE programme content, parents expressed fear and denial regarding the expression of sexuality. Young people with ID identified relationships and knowledge. ID professionals identified safety and legal issues. Health professionals identified health issues and concerns. Across the groups, all identified the need to involve care givers to enable them to support social, situational learning opportunities.	Evidence-based programmes need to be developed involving people with ID at all stages. Professionals need to build upon their experience of working with young people with ID. Further research is required to identify whether sex education programmes decrease the risk of sexual abuse and exploitation. Rigorous RSE programme evaluations are needed to identify effectiveness and outcomes.
Wilson & Frawley (2016) Australia	Identify the support offered to young people with intellectual and developmental disability (IDD).	Transition to work staff (TTW) to include sexuality and relationship information in programmes.	Support staff (*n* = 17)	Qualitative: focus groups using thematic analysis	Some support workers felt perceived as ‘reluctant counsellors’. Participants felt poorly prepared to deliver and discuss sex education and sexuality issues and relied on their own attitudes and values to guide their practice. Possibility of ‘blurred’ lines between education and social support role.	Further research is needed regarding policy and practice development to inform RSE programme design, delivery and evaluation to identify effectiveness and outcomes.
